# The Costs and Cost-Effectiveness of a District-Strengthening Strategy to Mitigate the 3 Delays to Quality Maternal Health Care: Results From Uganda and Zambia

**DOI:** 10.9745/GHSP-D-18-00429

**Published:** 2019-03-11

**Authors:** Benjamin Johns, Peter Hangoma, Lynn Atuyambe, Sophie Faye, Mark Tumwine, Collen Zulu, Marta Levitt, Tannia Tembo, Jessica Healey, Rui Li, Christine Mugasha, Florina Serbanescu, Claudia Morrissey Conlon

**Affiliations:** a International Development Division, Abt Associates Inc., Bethesda, MD, USA.; b Department of Health Policy and Management, School of Public Health, University of Zambia, Lusaka, Zambia.; c Department of Community Health and Behavioral Sciences, School of Public Health, College of Health Sciences, Makerere University, Kampala, Uganda.; d Uganda Country Office, U.S. Centers for Disease Control and Prevention, Entebbe, Uganda.; e U.S. Agency for International Development, Lusaka, Zambia.; f Bureau for Global Health, U.S. Agency for International Development, Washington, DC, USA, and RTI, Washington, DC, USA. Now with Palladium, Abuja, Nigeria.; g Centre for Infectious Disease Research in Zambia, Lusaka, Zambia.; h U.S. Agency for International Development, Lusaka, Zambia. Now based in Monrovia, Liberia.; i Division of Reproductive Health, U.S. Centers for Disease Control and Prevention, Atlanta, GA, USA.; j U.S. Agency for International Development, Kampala, Uganda.; k Bureau for Global Health, U.S. Agency for International Development, Washington, DC.

## Abstract

The primary objective of this study was to estimate the costs and the incremental cost-effectiveness of maternal and newborn care associated with the Saving Mothers, Giving Life (SMGL) initiative—a comprehensive district-strengthening approach addressing the 3 delays associated with maternal mortality—in Uganda and Zambia. To assess effectiveness, we used a before-after design comparing facility outcome data from 2012 (before) and 2016 (after). To estimate costs, we used unit costs collected from comparison districts in 2016 coupled with data on health services utilization from 2012 in SMGL-supported districts to estimate the costs before the start of SMGL. We collected data from health facilities, ministerial health offices, and implementing partners for the year 2016 in 2 SMGL-supported districts in each country and in 3 comparison non-SMGL districts (2 in Zambia, 1 in Uganda). Incremental costs for maternal and newborn health care per SMGL-supported district in 2016 was estimated to be US$845,000 in Uganda and $760,000 in Zambia. The incremental cost per delivery was estimated to be $38 in Uganda and $95 in Zambia. For the districts included in this study, SMGL maternal and newborn health activities were associated with approximately 164 deaths averted in Uganda and 121 deaths averted in Zambia in 2016 compared to 2012. In Uganda, the cost per death averted was $10,311, or $177 per life-year gained. In Zambia, the cost per death averted was $12,514, or $206 per life-year gained. The SMGL approach can be very cost-effective, with the cost per life-year gained as a percentage of the gross domestic product (GDP) being 25.6% and 16.4% in Uganda and Zambia, respectively. In terms of affordability, the SMGL approach could be paid for by increasing health spending from 7.3% to 7.5% of GDP in Uganda and from 5.4% to 5.8% in Zambia.

## Abstract (precis)

A comprehensive district-strengthening approach to address maternal and newborn health was estimated to cost US$177 per life-year gained in Uganda and $206 per life-year gained in Zambia. The approach represents a very cost-effective health investment compared to GDP per capita.

## INTRODUCTION

Sub-Saharan Africa has the highest lifetime risk of maternal mortality (1:36) of any region in the world.^1^^,^^2^ For example, the maternal mortality ratio in 2015 was 546 per 100,000 live births, with an estimated 201,000 maternal deaths. The maternal mortality ratio in sub-Saharan Africa is almost 22 times that in Europe.^1^^,^^2^ Studies have documented the financial, economic, and social consequences of maternal deaths, including increased risk of death for newborns and lower educational achievement, poorer economic outcomes, and poorer health for surviving children.^3^^,^^4^ Coverage of essential antenatal, maternal, and newborn health services remains below levels needed to reach internationally agreed upon goals.^5^ Despite these continuing challenges, the maternal mortality ratio declined in sub-Saharan Africa between 1990 and 2015 by 45%,^6^ coinciding with the scale-up of essential antenatal, maternal, and newborn interventions.

Three health system barriers have long been known to delay timely access to quality obstetric and newborn care (the “3 delays”): (1) barriers in deciding to seek care at a health facility; (2) barriers in reaching a facility in time to receive the needed care; and (3) barriers in receiving high-quality, respectful, and timely care at the facility.^7^ To reduce these barriers, stakeholders may implement an integrated package of supply-side interventions, particularly health system strengthening activities to ensure quality care, and demand-side interventions within and outside the health facility setting to increase knowledge of, access to, and utilization of care.^5^^,^^8^

Three health system barriers have long been known to delay timely access to quality obstetric and newborn care—the 3 delays pertain to seeking care at a health facility, reaching a facility in time, and receiving quality care once there.

The significant costs to households and communities of a maternal death are well documented.^3^ Existing literature suggests that essential maternal health interventions are highly cost-effective.^9^^,^^10^ For example, based on regional-level estimates from the World Health Organization's (WHO's) Choosing Interventions that are Cost-Effective (CHOICE) model, a full package of maternal care costs 36 International dollars (I$) per disability-adjusted life-year (DALY) averted in high disease burden countries in sub-Saharan Africa (compared to no maternal care).^9^ Similarly, according to the Bill and Melinda Gates Foundation-funded Disease Control Priorities project, emergency obstetric care costs US$10 per DALY averted in low- and middle-income countries.^10^ Thus, the cost per DALY averted for maternal care appears to be well below the average gross domestic product (GDP) per capita in any country in the world. However, these studies focus primarily on improvements in clinical care, which is associated with the third delay. A review conducted by the Disease Control Priorities project also contains primarily interventions based in health facilities.^11^ The WHO-CHOICE model lists community-based interventions for antenatal and neonatal care “including outreach,” but it does not specify what constitutes outreach.^9^

Other studies demonstrate the effectiveness of interventions to reduce one or more of the 3 delays,^12^^–^^14^ but the literature on the cost of these interventions is limited. A recent review of the costs of maternal care in low- and middle-income countries found 8 studies assessing the costs of antenatal care and 18 studies assessing the costs of delivery.^15^ Of these, only 1 study from sub-Saharan Africa included the costs of community-based maternal support.^15^ Further, existing literature on the cost-effectiveness of maternal health interventions tends to focus on the additional costs and effectiveness of a single intervention^16^^–^^20^ that typically addresses only 1 of the 3 delays. A few cost-effectiveness studies include health systems strengthening as a complement to a demand generation intervention^21^ or assess the cost-effectiveness of a more comprehensive approach to improving coverage of skilled care at birth.^22^ Overall, however, the literature assessing the costs and cost-effectiveness of a comprehensive health systems strengthening approach to address all 3 delays is scarce. An exception is a cost-effectiveness analysis of maternal and newborn interventions in Uganda under Phase 1 of the Saving Mothers, Giving Life (SMGL) initiative,^23^ but this study did not account for the full costs of interventions, chiefly because indirect facility overheads were not considered.

The primary objectives of the current study were to estimate the costs and incremental cost-effectiveness of maternal and newborn care associated with SMGL's comprehensive district- strengthening approach to addressing the 3 delays in selected districts in Uganda and Zambia. Secondarily, we assessed the sources of financing for the SMGL interventions. Findings from our analyses can inform stakeholder investments on cost-effective means to reduce maternal and perinatal mortality.

## CONTEXT AND SMGL INTERVENTIONS

Uganda and Zambia have very high maternal mortality levels, despite the occurrence of substantial reductions, including downward national trends before and during the period of SMGL implementation.^24^ The maternal mortality ratio in Zambia declined from 591 maternal deaths per 100,000 live births as measured in 2007^25^ to 398 per 100,000 live births as measured in 2013.^26^ The maternal mortality ratio in Uganda declined from 432 maternal deaths per 100,000 live births as measured in 2011^27^ to 336 per 100,000 live births as measured in 2016.^28^ The neonatal mortality rate in Uganda and Zambia in 2011–2013 was estimated to be 27 and 24 per 1,000 live births, respectively.^26^^,^^28^ In Uganda, 57% of women delivered in health facilities in 2011, including 52% of women in rural areas.^27^ In Zambia, about two-thirds of women delivered in health facilities in 2013; however, in rural areas, this percentage was 56%.^26^

Against this background, SMGL was implemented in 2012 in an effort to dramatically and rapidly reduce maternal mortality in selected districts of Uganda and Zambia (and later, Nigeria). The SMGL approach is based on context-specific solutions to maternal and, later, newborn health (MNH) problems. These solutions are identified and implemented through a coalition of partners, including governments, nongovernmental organizations, and the private sector.^29^ SMGL principles include the following:

1. Understanding the maternal health ecosystem in a given geographic area through formal assessment of both public and private sectors.
2. Using scarce resources rationally to address gaps and improve access to and quality of care.
3. Addressing all 3 delays to care to ensure access to lifesaving care within 2 hours of the onset of a complication, focusing on the period of labor, delivery, and the 72 hours postpartum when women and newborns are most vulnerable.^30^
4. Decreasing missed opportunities by integrating MNH and HIV services.
5. Counting, analyzing, and reporting all maternal and newborn deaths, and using findings to improve care.^24^

The SMGL approach is based on context-specific solutions to maternal and newborn health problems.

With substantial subnational variation in maternal mortality ratios within these countries, SMGL targeted districts that had among the poorest maternal health indicators in each country.^24^ The districts selected in Uganda were Kabarole, Kamwenge, Kibaale, and Kyenjojo. In Zambia, the districts selected were Kalomo, Lundazi, Mansa, and Nyimba.^24^

In the first year of implementation, SMGL was associated with a 35% reduction in the institutional maternal mortality ratio across the 2 countries.^7^ Donor investment was planned to be largest in the first years of the SMGL initiative, with national and local governments assuming greater responsibility for SMGL costs over time. Table 1 lists the interventions and activities included in the costing estimates for Uganda and Zambia.

**TABLE 1.** Activities and Interventions Included in the Costing Estimates

Activity or Intervention	Implemented in Uganda, Zambia, or Both^a^
Activities targeting delay 1^b^	
Train community groups (VHTs and SMAGs) to promote facility delivery and birth preparedness	Uganda and **Zambia**
Procure bicycles, equipment, and supplies for community groups	Uganda and **Zambia**
Provide financial support to community activities (e.g., funding to attend monthly meetings, supervision costs, community assessment mappings)	Uganda and Zambia
Produce a documentary about safe motherhood using traditional leaders	Zambia
Run mass media campaigns on safe motherhood (including development of materials, air time costs, and translation costs), engage community drama groups	Uganda and Zambia
Identify and engage community change champions in safe motherhood	Zambia
Provision of revolving Fund for Village Saving Schemes	Uganda
MNH outreach (project or community staff visits to communities)	Uganda and Zambia
Activities targeting delay 2^b^	
Distribution of subsidized vouchers for transport to delivery in EmONC facilities, public and private(transport to antenatal and postnatal care were added in Phase 2)	Uganda
Procurement of ambulances, motorcycles, and motorbikes for transportation and referrals	Uganda and **Zambia**
District-level transport committees to improve referral	Uganda
Renovate MWHs near hospitals for high-risk women	Uganda and Zambia, primarily Zambia
Train MWH staff to operate maternity homes; costs and revenue from income-generating activities; provision of food for those in maternity homes (as applicable)	Zambia
Activities targeting delay 3^b^	
Provide antenatal care	**Uganda and Zambia**
Provide basic delivery care	**Uganda and Zambia**
Provision of comprehensive emergency care (blood transfusion/cesarean delivery)	**Uganda and Zambia**
Upgrade care in neonatal special care units, including purchase of equipment, training, and provision of essential medicines	**Uganda** and Zambia
Increase facility EmONC capacity, including purchase of EmONC equipment and provision of essential medicines	**Uganda** and Zambia
Establish/expand/refurbish maternity blocks, neonatal special care units, laboratories, pharmacies, and operating theaters	Uganda and Zambia
Hire new doctors, nurses, and midwives	Uganda and Zambia, primarily Uganda
Train health workers in essential newborn care and neonatal resuscitation	Uganda and Zambia
Train doctors in surgical obstetric care and nurses in anesthesia, train/mentor nurses in basic EmONC	Uganda and Zambia
Other training and mentoring (e.g., rapid syphilis screening, PMTCT, essential newborn care, UBT, maternal and perinatal death reviews)	Uganda and Zambia; UBT in Zambia
Supervision of frontline workers to maintain/improve skills in obstetrics/newborn care	Uganda and Zambia
Provide essential medicines	**Uganda and Zambia**
Provide training and oversight for maternal death reviews	Uganda and Zambia
Conduct health facility assessments	Uganda and Zambia
Health systems strengthening and program management^c^	
Strengthen supply chain through training on procurement and stock management	Uganda and Zambia
Build capacity of facility staff to supervise community health workers (first delay)	Zambia
Provide computer-based medical records (SmartCare)	**Zambia**
Strengthen pharmacy, laboratory, and blood supply	Uganda and Zambia
Train health workers in data collection and health information systems (DHIS2)	Uganda and Zambia
Strengthen program management (staff, vehicles, meetings, workshops, etc., including management of SMGL program, monitoring and evaluation, etc.) (above facility costs)	Uganda and Zambia
Build provincial and district health team capacity with SMGL-supported staff (above facility costs)	Uganda and Zambia

Abbreviations: DHIS2, district health information system 2; EmONC, emergency obstetric and neonatal care; MNH, maternal and newborn health; MWH, maternity waiting home; PMTCT, prevention of mother-to-child transmission; SMAG, Safe Motherhood Action Group; SMGL, Saving Mothers, Giving Life; UBT, uterine balloon tamponade; VHT, Village Health Team.

a In countries shown in boldface, the activities were conducted in both SMGL and comparison districts, although frequently at lower intensity/scale in comparison districts than in SMGL districts. Source: Interviews with implementing partners and district and provincial health office staff.

b Primary delay addressed refers to which of the 3 delays the activities is assumed to mainly address (since some of the inputs/activities may address more than one).

c Categorized as primarily addressing the third delay unless otherwise noted.

## METHODOLOGY

### Study Design

We calculated the costs per maternal death averted and life-year gained by combining data on intervention costs that we compiled with direct outcome evaluation data from studies that previously documented maternal and newborn mortality associated with the SMGL approach. Health impact data in the SMGL-supported districts were collected in a separate evaluation of SMGL.^30^^,^^31^ The impact evaluation used a before-after design comparing selected health indicators and outcomes in 2012 (baseline) and 2016 (endline). These evaluations, including the data sources and the impact results, are described elsewhere in this special supplement.^30^

The estimated costs of MNH interventions were assessed in a subset of the districts where the SMGL approach was implemented and compared to estimated costs in 2012, prior to SMGL interventions. Since costs were not directly collected in the SMGL-supported districts prior to SMGL implementation in 2012, we derived comparison costs from the 2016 unit costs (e.g., cost per antenatal care visit, cost per vaginal delivery, cost per cesarean delivery) in neighboring districts where MNH programs were chiefly supported by country government efforts alone, to be consistent with the time frame for the effectiveness evaluation (end year as 2016). Table 2 lists the variables used in the cost-effectiveness analysis, along with the sources of data.

**TABLE 2.** Parameters Used to Calculate District Costs of MNH Care, Life-Years Lost Due to Maternal Death, and Incremental Cost-Effectiveness of Deaths Averted

Number	Parameter	Value	Data Source	Notes
Costs (all)				
1	Discount rate	3%	WHO-CHOICE recommendation^34^	Locally published discount rates used in sensitivity analysis (15% in Uganda and 9.7% in Zambia)^35^^,^^36^
Costs 2012				
2	Costs associated with the first delay	Varies by district (see Table 4)	Interviews with health facility staff, district health office staff, provincial health office staff, and implementing partners in comparison districts	Interviews covered the period 2012 through 2016; start-up activities and capital costs were tracked. Costs for existing maternity waiting homes are included.
3	Costs associated with the second delay	Varies by district (see Table 4)	Interviews with health facility staff, district health office staff, provincial health office staff, implementing partners, and review of ambulance log books in comparison districts	Interviews covered the period 2012 through 2016; start-up activities and capital costs were tracked.
4	Unit cost of ANC	Varies by type of facility (see Table 3)	Data collection at health facilities in comparison districts, interviews with implementing partners	Inclusive of facility overhead costs
5	Number of ANC visits	Ratio of ANC visits to number of facility births	Data from health facility registers/district health offices in comparison districts	Number of facility births based on SMGL districts data from 2012
6	Unit cost of vaginal delivery	Varies by type of facility (see Table 3)	Data collection at health facilities in comparison districts, interviews with implementing partners	Inclusive of facility overhead costs and admissions (for mother and newborn)
7	Number of vaginal deliveries	Varies by district	Data from health facility registers/district health offices in comparison districts, Serbanescu and colleagues^30^	Number for SMGL districts in 2012
8	Unit cost of cesarean delivery	Varies by type of facility (see Table 3)	Data collection at health facilities in comparison districts, interviews with implementing partners	Inclusive of facility overhead costs and admissions (for mother and newborn)
9	Number of cesarean deliveries	Varies by district	Data from health facility registers/district health offices in comparison districts, Serbanescu and colleagues^30^	Number for SMGL districts in 2012
10	Above community/facility costs	Varies by district (see Table 4)	Interviews with health facility staff, district health office staff, provincial health office staff, and implementing partners in comparison districts	Interviews covered the period 2012 through 2016; start-up activities and capital costs were tracked.
11	Total costs of MNH care in 2012	Calculation	Based on parameters 2–10	
Costs 2016				
12	Costs associated with the first delay	Varies by district (see Table 4)	Interviews with health facility staff, district health office staff, provincial health office staff, and implementing partners in SMGL districts	Interviews covered the period 2012 through 2016; start-up activities and capital costs tracked. Costs for maternity waiting homes are included.
13	Costs associated with the second delay	Varies by district (see Table 4)	Interviews with health facility staff, district health office staff, provincial health office staff, implementing partners, and review of ambulance log books in SMGL districts	Interviews covered the period 2012 through 2016; start-up activities and capital costs were tracked.
14	Unit cost of ANC	Varies by type of facility (see Table 3)	Data collection at health facilities in SMGL districts, interviews with implementing partners	Inclusive of facility overhead costs
15	Number of ANC visits	Ratio of ANC visits to number of facility births	Data from health facility registers/district health offices in SMGL districts	Number of facility births based on SMGL districts data from 2016
16	Unit cost of vaginal delivery	Varies by type of facility (see Table 3)	Data collection at health facilities in SMGL districts, interviews with implementing partners.	Inclusive of facility overhead costs and admissions (for mother and newborn)
17	Number of vaginal deliveries	Varies by district	Serbanescu and colleagues^30^	Number for SMGL districts in 2016
18	Unit cost of cesarean delivery	Varies by type of facility (see Table 3)	Data collection at health facilities in SMGL districts, interviews with implementing partners	Inclusive of facility overhead costs and admissions (for mother and newborn)
19	Number of cesarean deliveries	Varies by district	Data from health facility registers/district health offices in SMGL districts, Serbanescu and colleagues^30^	Number for SMGL districts in 2016
20	Above community/ facility costs	Varies by district (see Table 4)	Interviews with health facility staff, district health office staff, provincial health office staff, and implementing partners in comparison districts	Interviews covered the period 2012 through 2016; start-up activities and capital costs were tracked.
21	Total costs of MNH care in 2016	Calculation	Based on parameters 12–20	In Uganda, included cost of patients referred to Fort Portal referral hospital
Deaths in 2012				
22	Number of facility-based deliveries	Varies by district	POMS and unpublished district data,^31^ district offices in SMGL districts	Number of deliveries for SMGL districts in 2016 multiplied by the institutional delivery rate in 2012
23	Maternal death ratio	534 deaths (Uganda) and 370 deaths (Zambia) per 100,000 live births	Serbanescu and colleagues^30^	
24	Perinatal death rate	39.3 (Uganda) and 37.9 deaths (Zambia) per 1,000 births	Serbanescu and colleagues^30^	
25	Number of maternal deaths	Calculation	Parameter 22 × proportion of deliveries with live births/100,000 × Parameter 23	
26	Number of perinatal deaths	Calculation	Parameter 22/1,000 × Parameter 24	
27	Total number of deaths	Calculation	Parameter 25 + Parameter 26	
28	Life-years lost due to death	Years of life left estimated as 62.5 and 45.6 for perinatal and maternal death in Uganda and 62.3 and 45.7 for perinatal and maternal death in Zambia	WHO life tables^40^^,^^41^	Assume average age at death for maternal death is 27.5, for perinatal in first 2 days of life
Deaths in 2016				
29	Number of facility-based deliveries	Varies by district	POMS and unpublished district data,^31^ district offices in SMGL districts	Number for SMGL districts in 2016; varied in sensitivity analysis based on results for all SMGL districts^24^
30	Maternal death ratio	300 deaths (Uganda) and 231 deaths (Zambia) per 100,000 live births	Serbanescu and colleagues^30^	Decreased the percentage reduction in deaths results by 10 percentage points in sensitivity analysis
31	Perinatal death rate	34.4 (Uganda) and 28.2 deaths (Zambia) per 1,000 births	Serbanescu and colleagues^30^	
32	Number of maternal deaths	Calculation	Parameter 29 × proportion of deliveries with live births/100,000 × Parameter 30	
33	Number of perinatal deaths	Calculation	Parameter 29/1,000 × Parameter 31	
34	Total number of deaths	Calculation	Parameter 32 + Parameter 33	
35	Life-years lost due to death	Years of life left estimated as 62.5 and 45.6 for perinatal and maternal death in Uganda and 62.3 and 45.7 for perinatal and maternal death in Zambia	WHO life tables^40^^,^^41^	Assume average age at death for maternal death is 27.5, for perinatal in first 2 days of life. Years of life left estimated as 62.5 and 45.6 for perinatal and maternal death in Uganda and 62.3 and 45.7 for perinatal and maternal death in Zambia.
Incremental cost-effectiveness				
36	Incremental costs	Calculation	Parameter 21 − Parameter 11	In sensitivity analysis, reassess with all donor costs treated as incremental costs.
37	Incremental deaths averted	Calculation	Parameter 34 − Parameter 27	
38	Incremental life-years gained	Calculation	Parameter 35 − Parameter 28	
39	Incremental cost per death averted	Calculation	Parameter 36/Parameter 37	
40	Incremental cost per life-year gained	Calculation	Parameter 36/Parameter 38	

Abbreviations: ANC, antenatal care; MNH, maternal and newborn health; POMS, Pregnancy Outcome Monitoring Survey; SMGL, Saving Mothers, Giving Life; WHO CHOICE, World Health Organization's Choosing Interventions that are Cost-Effective.

The estimated costs of MNH interventions were assessed in selected districts where the SMGL approach was implemented and compared to estimated costs in 2012, prior to SMGL interventions.

Because cost data prior to SMGL implementation were not available, we assumed that SMGL would affect the unit cost of health services through both the scale (quantity utilized) and quality of services.^32^ Thus, we selected comparison districts in the costing analysis for proxy measures of costs before SMGL, assuming that the unit prices of health services in these districts were similar to those in the SMGL-supported districts prior to interventions. We also assumed that unit costs in the comparison districts did not change substantially during the 2012–2016 time period, and we conducted sensitivity analyses to explore this assumption. We used districts as the unit of analysis because the SMGL approach was implemented at the district level, and many of the costs were incurred at the district level and could not be easily attributed to specific health facilities. This study assessed costs associated with provision of MNH care retrospectively in the SMGL intervention districts for the year 2016, including annualized start-up costs and capital costs over the 2012–2016 period. Start-up costs are defined as the costs for activities needed to establish interventions that are not incurred on an annual basis, while capital costs include the purchase of durable goods that are used over multiple years. Thus, we assumed effects are not cumulative across years except to the extent that continued capacity building, which is captured in start-up costs, allowed for increasing the effectiveness of the SMGL approach over time. We also assessed unit costs in comparison districts for the year 2016. We then used these unit costs together with 2012 utilization data from SMGL intervention districts to estimate costs in 2012 in the SMGL-supported districts (before SMGL started).

### Selection of Study Areas

Planned data collection included 2 of the 4 learning districts in each country. In Zambia, we randomly selected Mansa and Nyimba from the 4 SMGL-supported districts for inclusion in these analyses. Mansa became 2 separate districts (Mansa and Chembe) in 2012, and data were collected from both. We also selected 2 districts, Kapiri Mposhi and Mbala, for comparative purposes for the costing analyses. The intent was to conduct the costing study in districts used in an external evaluation of SMGL at the end of Phase 1, where 2 comparison districts were selected to be similar to SMGL-supported districts across a number of factors (including health infrastructure, geography and climate, health utilization, morbidity and mortality, and socioeconomic context) that would also likely influence costs.^29^ However, one of the comparison districts (Kabwe) later received extensive donor support for MNH programs and was excluded. We decided to select a second comparison district from the Northern Province. We randomly selected Mbala district after excluding districts participating in the World Bank's results-based financing project in the province.

In Uganda, we purposively included the greater Kibaale district (now existing as 3 districts; data were collected from all 3 districts) in the study because it was the only SMGL district to receive extensive support from one of the 2 main implementing partners. From the other 3 SMGL-supported districts, we selected Kyenjojo as the remaining rural district with a district hospital. Both districts received similar SMGL-supported interventions, with the exception of transport vouchers, which were implemented in Kyenjojo only.^33^ We also included the Fort Portal Referral Hospital, which received referrals from both Kibaale and Kyenjojo (and is located in a third SMGL district). For comparison purposes, we selected Masindi district, which served as a comparison district in an early evaluation of SMGL because it is located in the Western region and has a population and health system similar to the SMGL-supported districts. Due to limited time and budget, only one comparison district was included in Uganda.

In each of the selected districts of Uganda and Zambia, we collected data from the district health office, the government hospital in that district, and 2 randomly selected government health centers. Overall, we collected data from 5 districts, 4 hospitals, and 6 health centers in Uganda (3 health centers level III and 3 health centers level IV—one of each type in each of 2 SMGL-supported districts and in the comparison district), and 5 districts, 4 hospitals, and 8 health centers in Zambia. Data relating to MNH care activities in these districts were collected from district and provincial health offices (where appropriate) at the national level and from implementing partners, including 3 implementing partners in Uganda and over 30 in Zambia involved in MNH care, in the districts included in this analysis.

Overall, we collected data from 5 districts, 4 hospitals, and 6 health centers in Uganda, and 5 districts, 4 hospitals, and 8 health centers in Zambia.

### Ethical Approval

The data collection specific to this study was exempted from the need for ethical approval by Abt Associates Institutional Review Board and from the University of Zambia Biomedical Research Ethics Committee in July 2017 because it did not include research on human subjects. The study received approval from the Makerere University of Public Health Higher Degrees, Research, and Ethics Committee in January 2018 and the Uganda National Council for Science and Technology (approval number SS 4511) in February 2018.

### Data Collection

Data collection at health facilities occurred in July–August 2017 in Zambia and February–March 2018 in Uganda. Trained data collection teams extracted information on health facility area (square meters), staffing, service utilization, vehicles, and consumption of commodities from these facilities. Data were entered into Microsoft Excel templates designed for the study and were reviewed daily by data collection supervisors and again by the research team. Questions were sent to data collectors to verify information, and facilities were contacted again to clarify ambiguous information as needed. Similarly, structured templates were used to capture data at district health offices and, where appropriate, provincial health offices related to overall district health statistics (e.g., number of deliveries, number of health facilities) and activities related to MNH (e.g., training, health systems strengthening, mentoring, supervision, community outreach) during 2012–2016 for annualized start-up costs.

Data collection templates were constructed based on past analyses of expenditures in SMGL areas^34^^,^^35^ and sent to implementing partners. Data collectors then visited these partners to provide support for extracting the necessary data. Implementing partners provided data on all relevant start-up activity costs, capital expenditures, and routine activities for 2012–2016. Costs for national-level activities were not included unless the activity specifically focused on one of the districts included in these analyses; thus, for example, these analyses did not include costs for international staff and national staff working on multiple projects in addition to SMGL or costs for offices outside the SMGL districts.

Data were collected for all activities supporting MNH, whether or not they were “officially” part of SMGL. However, some activities were not assessed as part of the SMGL evaluation, including HIV/AIDS care or prevention for pregnant women and postpartum family planning outside the MNH clinics of health facilities, unless the SMGL program specifically included them.

Data were collected for all activities supporting MNH, whether or not they were “officially” part of SMGL.

### Data Analysis Methods

We used a 1-year analytic horizon for deaths averted (i.e., the difference between deaths occurring in 2012 and in 2016) and a lifetime analytic horizon for life-years gained. We included costs from the health system perspective—that is, costs incurred by the health system and implementing partners. Costs incurred by patients and volunteers' time were not captured or included in the analysis. Capital items and start-up costs were converted into annual equivalent costs using standard formulas; we assumed a 3% discount rate^36^ but explored locally published discount rates in sensitivity analyses.^37^^,^^38^ All costs were inflated to 2016 based on local GDP inflators and are presented in US dollars.^39^

We used a financial approach to estimate the costs of activities presented in Table 1. Costs of activities targeting communities included the support of volunteer community groups (Village Health Teams in Uganda and Safe Motherhood Action Groups in Zambia), including costs to train, equip, and organize group meetings.

We employed a mix of top-down and bottom-up costing methods to estimate health facility costs.^40^ Costs of administrative and support services (e.g., cleaning, maintenance) were allocated to maternal, newborn, and antenatal wards or clinics based on number of staff, size, number of prescriptions, or service utilization in a top-down manner. Costs for ambulances were allocated to MNH services based on a review of ambulance logs. Whenever possible, quantities of consumables used directly in provision of maternal and newborn care were estimated from existing registers and stock cards specifying the amounts issued to a ward or clinic. If these data were not available, we relied on either allocation based on utilization (for general drugs and supplies) or health facility staff opinion (for drugs used specifically for maternal health). Quantities of consumables were multiplied by their unit prices, which were collected at the national level. Staff costs, inclusive of salary and benefits, were allocated to MNH services based on assigned duty stations, opening hours, work patterns, and service utilization. In Uganda, costs for utilities and building costs in public facilities were estimated based on implementing partners' accounts of costs for similar items; in Zambia, costs for utilities and buildings were estimated from previous costing exercises (R Homan, FHI 360, written communication, January 2018). Almost 40% of delivering facilities in Uganda SMGL-supported districts and 9% in Zambia are private.^30^ Costs for maternal and newborn services at nongovernmental health facilities in Uganda were based on a previous study carried out in the same districts.^41^ Costs incurred at health facilities and reported by implementing partners were cross-checked to ensure that items were not double counted.

Total costs for MNH services for entire districts were estimated using the average unit costs from sampled facilities for different types of services (e.g., antenatal care, vaginal delivery, cesarean delivery) and multiplying the results by the total utilization of these services in a district. These figures include costs for inpatient admissions. For estimates of costs before the start of SMGL, we used utilization numbers from the SMGL-supported districts in 2012 and unit costs from comparison districts, while for 2016 we used utilization numbers and unit costs from SMGL-supported districts in 2016. We disaggregated these calculations by type of health facility. We then added costs incurred at the community level and “above service delivery costs” (e.g., costs for offices located in districts, general and office support staff, program vehicles, and other general management and planning activities) to the facility-based costs. For SMGL districts, the programmatic costs and facility costs to address the first, second, and third delay total costs were added to derive the total costs for the districts. To convert costs incurred outside health facilities in comparison districts to costs incurred in 2012, we divided these costs by the number of facility deliveries in the comparison districts and then multiplied the results by the number of facility deliveries estimated to have occurred without SMGL in the SMGL districts. This calculation assumes that costs outside facilities varied directly with the number of deliveries at facilities; however, costs outside facilities in the comparison districts were a small proportion of all costs. For the 2012 cost estimates, we included observed community-level and above service delivery level costs from the comparison districts, under the assumption that these activities also likely existed in SMGL-supported districts before the start of SMGL. We calculated costs per facility delivery in 2012 (i.e., baseline facility delivery costs) and in 2016 (defined as the costs of improved facility delivery, which included all costs associated with delivery, demand generation, and transport). Incremental costs were calculated by taking the difference between the estimated total costs in 2016 and those in 2012. Sources of financing (donor, government, and private) were tracked throughout this exercise.

To estimate the health impact, we used the facility-based maternal mortality ratios and perinatal death rates in 2016 in SMGL-supported districts multiplied by the reported number of facility-based deliveries for the cohort of women giving birth in 2016 to determine the number of deaths in 2016.^31^ To estimate the number of deaths that would have occurred in the same districts in the absence of SMGL, we started with the number of deliveries for SMGL districts in 2016 multiplied by the institutional delivery rate in 2012 to estimate the number of facility-based deliveries that would have occurred without SMGL. To account for secular trends in maternal mortality and perinatal deaths, we adjusted the facility-based maternal mortality ratios and perinatal death rates from 2012 by subtracting the change in these indicators at a national level from 2012 to 2016 from the SMGL district-specific 2012 figures (see Supplement 1).^27^^,^^28^^,^^42^^,^^43^ To reflect a generalizable cost-effectiveness applicable as broadly as possible, we used the facility-based death ratios/rates from all SMGL areas. However, since we collected costs in only half of the SMGL-supported districts, this approach assumes that costs in the districts included in the costing data collection did not differ substantially from those in the SMGL-supported districts where cost data were not collected. We subsequently explored this assumption in a sensitivity analysis (described below).

The number of deaths averted was estimated by subtracting the number of deaths in 2012 from the number of deaths in 2016. We estimated the incremental cost-effectiveness by dividing the incremental costs by the number of deaths averted in SMGL areas. Additionally, we estimated the potential remaining years of life for pregnant women and newborns at the time of death and calculated the cost per life-year gained using national life-expectancy data.^44^^,^^45^ For averted perinatal deaths, we calculated the cost per life-year gained assuming that stillborn infants and newborns would have had a full life expectancy.^46^

### Sensitivity Analyses

We conducted sensitivity analyses to explore the potential impact of our assumptions on the results. Sensitivity analyses were done by changing input variable amounts and assessing how results were altered. The following scenarios and variables were considered for sensitivity analyses:

1. We used locally published discount rates to calculate annual equivalent costs.
2. We re-estimated the increased number of deliveries at health facilities using data from all 4 SMGL-supported districts rather than the 2 districts included in the costing. Chance variation in the increase in the number of deliveries at health facilities between districts may change the results.
3. We re-estimated the proportion of facility deliveries by cesarean delivery, for the same reason as above and using the same method.
4. We re-estimated incremental costs by considering all donor-supported costs as incremental costs (as opposed to using estimated 2012 costs). Although donor funds may displace some other sources of funding, this provides an upper-end estimation of the incremental costs in the absence of other district data.
5. We re-estimated the cost of preventing a year of lost life considering a discount rate of 3% for future life-years, as suggested by the WHO-CHOICE guidance.^36^

We conducted sensitivity analyses to explore the potential impact of our assumptions on the results.

Applying each of these 5 scenarios, we also calculated a “combined-case” scenario in which all the above scenarios were included at the least favorable value. Finally, we re-ran the analyses using mortality rates/ratios specific to the 2 SMGL-supported districts included in the costing.

## RESULTS

### Unit Costs

Average unit costs of a vaginal delivery in facilities in SMGL districts were lower or comparable to costs in facilities in non-SMGL districts in Uganda in 2016 (Table 3). The opposite is true for Zambia, where average unit costs were generally higher in facilities in SMGL districts. Specifically, in Uganda facility-based cost (excluding training of staff) for a vaginal delivery ranged from $24 to $45 across types of facilities in districts where SMGL was implemented, compared to $25 to $57 across types of facilities in the comparison district. In Zambia, the cost of a vaginal delivery was $42 at health centers and $118 at hospitals (on average across types of hospitals) in districts where SMGL was implemented, compared to $18 and $56, respectively, in comparison districts.

**TABLE 3.** Average Unit Cost of Selected Services at Health Facilities in 2016

	Uganda		Zambia	
	SMGL-Supported Districts	Comparison District	SMGL-Supported Districts	Comparison Districts
Vaginal delivery				
Health center III	$41	$42		
Health center IV	$45	$57		
Health center			$42	$18
District/general hospital	$26	$25	$12	$28
Referral hospital	$24	Not available	$125	$112
Cesarean delivery				
Health center IV	$202	$337		
District/general hospital	$163	$140	$33	$616
Referral hospital	$79	Not available	$495	$458
Antenatal care visit				
Health center III	$3.66	$5.49		
Health center IV	$3.59	$5.07		
Health center			$4.50	$3.96
District/general hospital	$5.03	$4.60	$6.96	$10.75
Referral hospital	$4.92	Not available	$38.90	Not available

Abbreviations: SGML, Saving Mothers, Giving Life.

Notes: The table includes only costs incurred at the facility level; it does not include training of facility staff. Results are presented in US 2016 dollars inclusive of capital and facility overhead costs. Data were not collected from the referral hospital receiving cases from Masindi.

Similarly, cesarean delivery unit costs in Uganda were lower in health centers ($202) in SMGL districts than in health centers in the comparison district ($337). However, the costs were higher in Uganda hospitals ($163) in SMGL districts than in hospitals ($140) in the comparison district. At the referral hospital in an SMGL district, the cost of a cesarean delivery was $79 because the operating theater had a relatively high volume of services. In Zambia, the average cesarean delivery unit costs were lower in hospitals ($468 on average across types of hospitals) in SMGL districts compared to hospitals ($508) in comparison districts.

Average unit costs of an antenatal care visit were lower in health centers ($3.66 for level III and $3.59 for level IV) in SMGL districts than in health centers ($5.49 for level III and $5.07 for level IV) in the comparison district in Uganda. In contrast, hospitals in SMGL districts had higher average unit costs ($5.03) compared to hospitals ($4.60) in the comparison district for an antenatal visit. The cost structure in Zambia was different; health centers in SMGL districts had higher average unit costs of an antenatal care visit ($4.50) than facilities ($3.96) in comparison districts, while there were mixed results from the comparison of SMGL hospitals and non-SMGL hospitals.

Each improved facility delivery cost about $104 in Uganda and $196 in Zambia.

### Total Costs

In 2012 in Uganda, total costs for MNH care were estimated to be about $650,000 per district, or almost $66 per facility delivery, while total costs for MNH care in Zambia in 2012 were estimated to be just under $425,000 per district or about $101 per facility delivery. Total costs for MNH care per district for the year 2016 were approximately $1.5 million in SMGL-supported districts in Uganda and almost $1.2 million in Zambia (Table 4). This translates to approximately $104 per “improved facility delivery” in Uganda and $196 per improved facility delivery in Zambia.

**TABLE 4.** Total Costs Per District and Sources of Financing

	Estimated Total Costs^a^ per SMGL District		Sources of Financing^a^					
			SMGL-Supported Districts			Comparison Districts		
	2016	2012	Government	Donor	Private	Government	Donor	Private
Uganda								
Costs associated with:								
The first delay	$300,422	$0	0%	100%	0%	100%	0%	0%
The second delay	$58,165	$40,123	2%	98%	0%	100%	0%	0%
The third delay	$983,364	$613,329	48%	27%	24%	94%	3%	3%
Above community/facility costs^b^	$156,931	$0	0%	100%	0%	100%	0%	0%
Total cost	$1,498,881	$653,452	35%	49%	16%	96%	2%	2%
Average number of facility deliveries	*14,419*	*9,947*						
Total cost per facility delivery	*$103.95*	*$65.70*						
Zambia								
Costs associated with:								
The first delay	$116,590	$7,608	10%	90%	N/A	52%	48%	N/A
The second delay	$107,149	$10,239	40%	60%	N/A	100%	0%	N/A
The third delay	$799,081	$405,234	74%	26%	N/A	97%	3%	N/A
Above community/facility costs^b^	$161,593	$1,663	0%	100%	N/A	100%	0%	N/A
Total cost	$1,184,413	$424,744	55%	45%	N/A	97%	3%	N/A
Average number of facility deliveries	*6,044*	*4,194*						
Total cost per facility delivery	*$195.98*	*$101.27*						

Abbreviations: N/A, not applicable; SMGL, Saving Mothers, Giving Life.

a Results are presented in US 2016 dollars, with capital and start-up costs converted to annual equivalent costs.

b Includes costs for offices located in districts, general and office support staff, program vehicles, and other general management and planning activities.

In 2016, donors covered about 49% of the MNH costs in Uganda and 45% of costs in Zambia in the SMGL-supported districts. In comparison districts, donors covered 2% (Uganda) and 3% (Zambia) of all costs in 2016. Costs incurred at private facilities accounted for 16% of costs in SMGL-supported districts in Uganda (although we were not able to assess the amount of donor financial support for births at private facilities). Donors supported the majority of costs associated with the first and second delays and just under 30% of costs related to the third delay in SMGLsupported districts.

Donors supported the majority of costs associated with the first and second delays and just under 30% of costs related to the third delay in both countries in SMGL-supported districts.

### Incremental Costs

In Uganda, the cost per facility delivery in 2016 in the SMGL-supported index districts was $38 higher than in 2012. Over 35% of the incremental cost went to support activities addressing the first delay, about 44% was spent on issues related to the third delay, 2% was spent on issues related to the second delay, and the remainder was spent on above community/facility costs for program support.

Similarly, the cost per facility delivery in Zambia in SMGL-supported districts was about $95 more in 2016 than in 2012. Addressing the first delay accounted for about 14% of the incremental cost, and above community/facility costs for program support were associated with approximately 21% of the incremental cost per facility delivery. About 52% of the incremental cost in SMGL-supported districts in Zambia addressed the third delay.

### Incremental Effects

In Uganda SMGL areas, the institutional maternal mortality ratio was 534 deaths per 100,000 live births in 2012 and 300 in 2016. The institutional perinatal morality rate was 39.3 per 1,000 births in 2012 and 34.4 in 2016 in SMGL areas. The percentage of deliveries in facilities changed from 45.5% in 2012 to 66.8% in 2016, and the population cesarean delivery rate increased from 5.3% to 9.0%.^30^

In Zambia SMGL areas, the institutional maternal mortality ratio declined from 370 deaths to 231 deaths per 100,000 live births and the institutional perinatal morality rate declined from 37.9 to 28.2 deaths per 1,000 births from 2012 to 2016. The percentage of deliveries in facilities increased from 62.6% to 90.2% and the population cesarean delivery rate increased from 2.7% to 4.8%.^30^

### Incremental Cost-Effectiveness Ratios

Based on the number of facility deliveries in the 4 districts included in this analysis, scale-up of MNH activities is associated with averting 164 deaths in Uganda and 121 deaths in Zambia in the 2 SMGL-supported districts in 2016 included in this analysis (Table 5). This translates to 9,549 years of life gained in Uganda and 7,362 years of life gained in Zambia. In Uganda, the incremental costs were estimated to be about $1,690,859, or $10,311 per death averted and $177 per life-year gained. With an estimated incremental cost of $1,519,338 in Zambia in 2016, the incremental cost per death averted was $12,514, or $206 per life-year gained.

**TABLE 5.** Incremental Cost-Effectiveness of SMGL in Uganda and Zambia

	Number of Facility Deliveries in 2016^a^	Number of Maternal Deaths^b^	Number of Perinatal Deaths^b^	Incremental Deaths Averted (Maternal and Perinatal)	Incremental Life-Years Gained	Total Cost^c^	Incremental Cost^c^	Incremental Cost per Death Averted^c^	Incremental Cost per Life-Year Gained^c^
Uganda									
Without SMGL	19,893	128	1,114			$1,306,904			
With SMGL	28,838	86	992	164	9,549	$2,997,763	$1,690,859	$10,311	$177
Zambia									
Without SMGL	8,839	40	450			$849,489			
With SMGL	12,087	28	341	121	7,362	$2,368,826	$1,519,338	$12,514	$206

Abbreviation: SGML, Saving Mothers, Giving Life.

a The number of district deliveries in 2016 multiplied by the institutional delivery rate for 2012 (for “without SMGL”) and for 2016 (for “with SMGL”) reported in Serbanescu et al.^30^

b Estimated using the 2016 facility deliveries with SMGL (for both “with SMGL” and “without SMGL”) and the total maternal/perinatal death rates for all SMGL-supported districts in 2016 (for with SMGL) and 2012^30^ with adjustments for national-level secular trends (see Supplement 1) to estimate deaths if SMGL had never occurred (for without SMGL).

c Results are presented in US 2016 dollars, and represent the totals for the 2 SMGL-supported districts included in the analyses.

### Sensitivity Analyses

**FIGURE 1:**
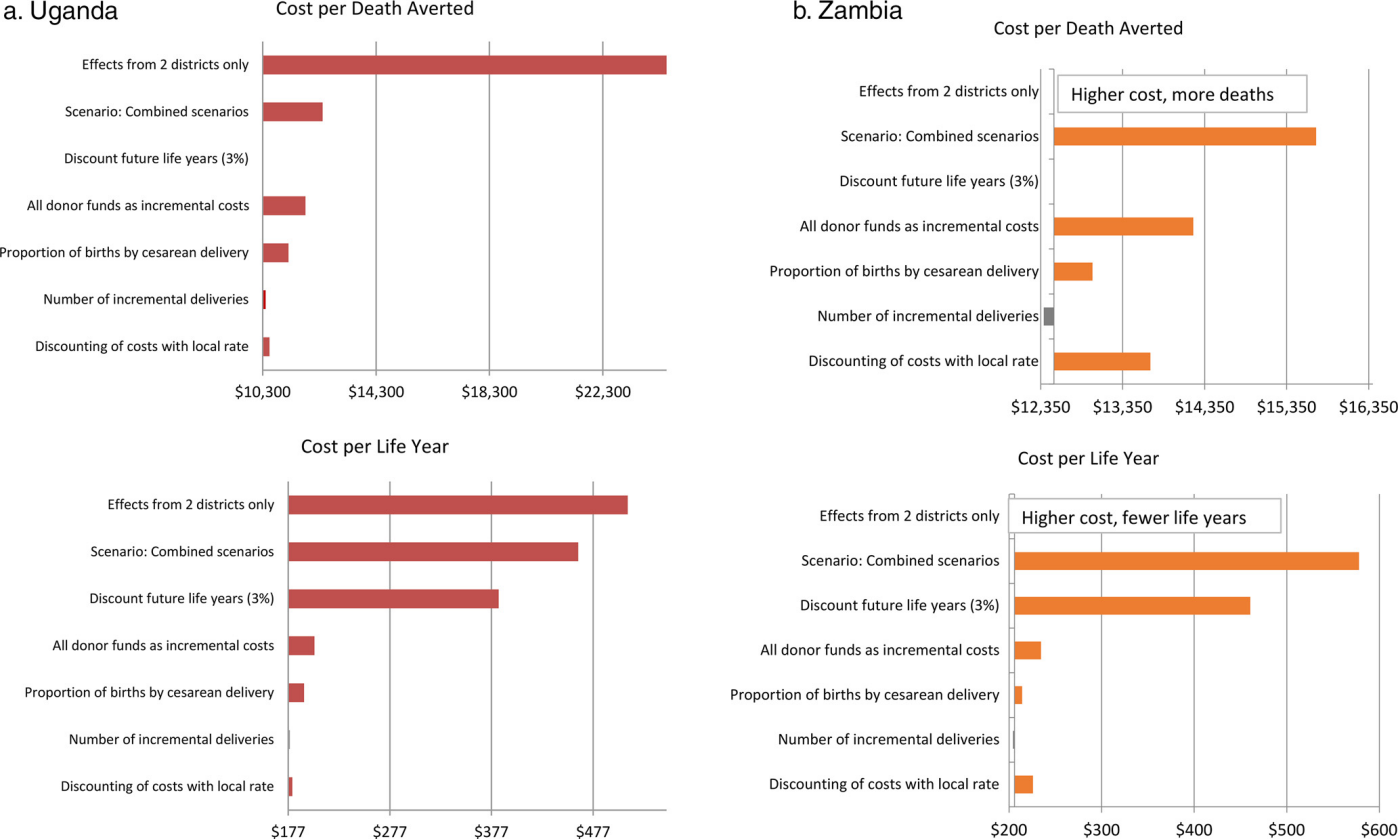
Results of Sensitivity Analysis for Uganda and Zambia

Figure 1a and Figure 1b depict for Uganda and Zambia, respectively, the cost per death averted or the cost per life-year gained along the x-axis, with each bar representing the change in the incremental cost-effectiveness ratio associated with changing an assumption. In Uganda, including effects only from Kibaale and Kyenjojo districts would result in a cost per death averted of about $25,550, with a cost per life-year gained of about $511 (Figure 1a). In Zambia, while the overall SMGL program was associated with reductions in mortality, using data from only Mansa and Nyimba resulted in a higher cost and reverse mortality effect (Figure 1b). This outcome was due to higher facility-based maternal and perinatal death rates in Mansa district in 2016 than in 2012, which were greater than the lower deaths rates in Nyimba district. The mortality increase in Mansa was largely due to more adverse outcomes that occurred in the referral hospital in Mansa in 2016, which provided delivery care to SMGL districts and to 5 additional non-SMGL-supported districts as well.

In Uganda and Zambia, results related to the cost per death averted were otherwise most sensitive to assumptions about using all donor costs as incremental costs. For any given scenario, the cost per death averted remained less than $12,000 in Uganda and $14,300 in Zambia. In a scenario combining the 5 main sensitivity analyses, where all assumptions were moved to the least favorable cost-effectiveness scenario, the cost per death averted was around $12,411 in Uganda and $15,708 in Zambia. The cost per life-year saved was most sensitive to the assumption about whether to discount future life-years. When future life-years were discounted, the cost per life-year gained increased to $384 in Uganda and $460 in Zambia. In the scenario combining the 5 main sensitivity analyses, the cost per life-year gained was $462 in Uganda and $578 in Zambia.

## DISCUSSION

In the 4 SMGL-supported districts included in these analyses, scale-up of MNH interventions prevented an estimated 285 institutional maternal and perinatal deaths in 2016, or about 71 death per district per year (0.6 death averted per 100 facility deliveries in Uganda and 1.0 death averted per 100 facility deliveries in Zambia). GDP per capita is a commonly used benchmark to determine whether or not an intervention is highly cost-effective, with the cost per DALY averted below the GDP per capita used as the benchmark for being highly cost-effective.^36^ The assessments of SMGL did not track changes in morbidity, and, to the extent that scale-up of MNH interventions prevented or ameliorated morbidity, our use of life-years gained likely underestimated the effects (as compared to DALYs averted).

In the 4 SMGL-supported districts included in these analyses, scale-up of MNH interventions prevented an estimated 285 institutional maternal and perinatal deaths in 2016.

Nevertheless, we found that the incremental cost per life-year gained in Uganda was $177, or 25.6% of the GDP per capita of $692, and the incremental cost per life-year gained in Zambia was $206, which is 16.4% of the GDP per capita of $1,257.

We found that the incremental cost per life-year gained was $177 in Uganda and $206 in Zambia.

A previous study assessing SMGL activities in Uganda suggested an incremental cost ranging from $28 to $104 per improved delivery, depending upon which activities were included in the costs, compared with our finding of about $38 per facility delivery.^23^ Another study assessing a maternal voucher scheme in Uganda, however, found that it cost about $340 per DALY averted, a higher ratio than we found here.^21^ However, only one district in our study promoted maternal vouchers, while a second had only 24% of facility deliveries supported by vouchers in 2016.^33^ Another study assessing surgical interventions for maternal health found a cost per DALY averted ranging from $7 to $360, depending on the procedure.^47^ Overall, the cost per life-year gained estimated here tends to be higher than the cost per DALY averted found in global models, but is similar to or lower than the cost per DALY averted from assessments of specific interventions in Uganda.

While recent estimates of unit costs of MNH activities are not available in Zambia, the unit costs found in this study are on the higher end of unit costs from other studies in Uganda. For example, a recent review found the cost of antenatal care in Uganda was about $5.90 at health centers and $6.40 at hospitals per woman,^15^ only marginally more than our estimated cost per antenatal care visit in Uganda. The same review also found that the cost per vaginal delivery in a facility in Uganda ranged from $5 to $46 across studies (compared with $24 to $45 in SMGL-supported districts and $25 to $57 in comparison districts documented here). The cost per cesarean delivery ranged from $61 to $108 (compared with $31 to $202 in SMGL-supported districts and $140 to $337 in comparison districts documented here).^15^ These findings suggest that the costs we estimated in our study are similar to or higher than those reported previously, at least for Uganda.

We did not see a marked change across the board in unit costs of services between SMGL-supported districts and comparison districts when we included only costs incurred at health facilities. In many cases, unit costs were lower in SMGL-supported districts. This was likely because of higher patient volumes in SMGL-supported facilities, with the increased efficiency in the use of capital and overhead costs offsetting the costs of increasing the quality of services. The exception was for vaginal delivery in Zambia, where unit costs were mostly higher in SMGL-supported districts than in comparison districts, but also where there was less difference in the number of deliveries between 2012 and 2016 than in Uganda. When we included costs incurred outside health facilities, including training, mentoring, and community mobilization—that is, the cost of an improved facility delivery—the cost per facility delivery in SMGL-supported districts was substantively higher than in comparison districts.

Funding for reducing the first delay constituted 36% of incremental costs in Uganda and 14% of incremental costs in Zambia, representing 20% and 10% of total costs in SMGL-supported districts, respectively. In comparison districts, the cost of activities addressing the first delay was either nonexistent (Uganda) or marginal ($1.81 per facility delivery in Zambia). Costs for the activities addressing the second delay were 4% and 9% of total costs in SMGL-supported districts in Uganda and Zambia, respectively. While funding for the second delay was similar in the SMGL-supported and comparison districts in Uganda (transportation vouchers were not implemented in Kibaale, and used only on a limited basis in 2016 in Kyenjojo), it was substantially higher in Zambia, where costs for maternity homes were a main cost driver for the second delay. For each facility delivery, $17.73 was spent on activities addressing the second delay in Zambia SMGL-supported districts, contrasting with $2.44 in comparison districts. The increase in costs per facility delivery was less marked (in percentage terms) for activities addressing the third delay, possibly representing either efficiencies, as noted above, or displacement of other funds. In terms of the total incremental costs, the third delay used the highest amount of resources in Uganda (about $370,000 per district) and in Zambia (about $394,000 per district). However, the results suggest that spending about 20% to 25% of MNH budgets to address the first 2 delays—critical delays that can prevent women from accessing care in a timely way—can be enough to improve receipt of timely facility care at birth. While securing and ensuring funding for activities to address the first 2 delays is critical, the results also suggest that in Uganda and Zambia, funding for facility deliveries was inadequate in 2012 to provide sufficient quantity and quality of care, with donors supporting more than 25% of costs addressing the third delay in SMGL districts in both Uganda and Zambia in 2016.

The results suggest that spending about 20% to 25% of MNH budgets to address the first 2 delays can be enough to improve receipt of timely facility care at birth.

This study is limited by use of comparison districts that were assessed only at the end of the SMGL program. These districts serve as an imperfect proxy estimate of the cost of MNH services before the start of the SMGL program. In addition, use of before and after data to estimate the effects of the scale-up of MNH services is subject to confounding due to secular trends. Although we tried to account for secular trends using national data, the national trends may not have been realized in the SMGL districts over the same time period.

Because data from 2016 in comparison areas were used as proxies for unit costs in SMGL-supported districts in 2012, we assessed data from 2016 in comparison districts with data from SMGL-supported districts in 2012 to ensure comparability. In Uganda, there were about 700 births per facility in SMGL-supported districts in 2012 and 500 births per facility in comparison districts in 2016, while in Zambia, there were about 225 births per facility in both 2012 in SMGL-supported districts and 2016 in comparison districts. In Uganda, 6% of facility births were by cesarean delivery in the SMGL-supported districts in 2012, compared with 9% in comparison districts in 2016, while in Zambia the percentage of facility births by cesarean delivery was 7% and 3%, respectively. Because data from comparison districts suggest a close match with intervention districts before the start of SMGL in some cases but a notable difference in other instances, we addressed potential biases in sensitivity analyses by using available data on likely ranges for changes in facility-based deliveries, cesarean deliveries, and incremental costs per facility delivery. In all cases, conclusions did not change substantively.

However, the effects presented here could potentially be underestimates for several reasons. Assessing progress in reducing facility maternal and perinatal mortality during the initiative required using facility data and data abstraction protocols. In 2012, each country faced the immediate challenge of how to produce baseline measurements of maternal and perinatal mortality in the period immediately before the initiative began and comparable measurements during the initiative, when data quality improvements were institutionalized. At baseline, each country used its existing data systems and infrastructure to devise its own independent data-collection approach. Although the definitions of indicators were standard, the quality of primary data used to calculate the number of maternal and perinatal deaths was substantially lower at baseline than at endline in both countries. In addition, differences in data collection existed between Uganda and Zambia. Thus, some deaths were likely missed in the baseline count, which would bias our results downward. Further, the proportion of deliveries in facilities increased over time, but we applied the facility-based death ratios/rates to all births. To the extent that women who would have given birth at home without SMGL would have worse outcomes than were observed for facility births, we underestimate the effects of the program.

Lacking data, we have not tried to incorporate these effects into the analyses. Further, the complete effects of the program, which may include increasing staff morale and their ability to deliver other interventions (such as family planning or prevention/elimination of mother-to-child transmission of HIV), were not captured in the effect estimates. While we did not assess changes in patient payments to access services, we also did not include the potential cost savings (from productivity losses and other social costs) resulting from preventing a maternal or newborn death.^3^^,^^48^

Donors spent upwards of $733,000 per district in Uganda and $538,000 per district in Zambia in total annual equivalent costs, and in the first year of SMGL $2 million and $1.5 per district in real budgetary expenditures. These findings are in keeping with a previous study assessing SMGL expenditures (the data from these studies were reviewed as part of these analyses).^34^^,^^35^ Recent global estimates suggest that $11 or more per capita per year in added costs are needed in sub-Saharan Africa to meet the full needs of MNH, sexual, and reproductive health care.^49^ While not achieving the full 80% mortality reductions suggested by the $11 per capita figure and including a different set of interventions, the incremental annual costs of the project represent about $1.36 per person living in the SMGL-supported districts in Uganda and $4.85 per person in Zambia. Thus, the SMGL project could be paid for by increasing health spending from 7.3% of GDP in 2015 (in Uganda) and 5.4% (in Zambia) to 7.5% and 5.8% of GDP, respectively.^44^^,^^45^ Further, SMGL used an accelerated and capital-intensive model in Uganda and Zambia. Excluding capital and start-up costs, the donor financing for recurrent costs in 2016 was about $645,000 per district in Uganda and $135,000 per district in Zambia—just over $1 per person in Uganda districts and about $0.86 per person in Zambia districts. The SMGL project utilized program implementation staff located in the SMGL-supported districts, the costs of which are included here. However, if the model is replicated, the cost structures the governments may use would possibly be different from those used by implementing partners, or some duplication of efforts may possibly be reduced. Thus, the 10% to 14% of costs represented by above service delivery and community costs could be reduced when the program is replicated. Further work assessing the future financial implications and budgetary impact of continuing SMGL (or implementing SMGL in other districts or countries) is needed.

While the results from Uganda and Zambia were similar in terms of their cost-effectiveness, the sensitivity analyses looking at results only for districts with cost data indicate that heterogeneity would certainly exist in applying the results to other settings and within countries themselves. SMGL was targeted to areas within Uganda and Zambia with high maternal mortality, with some activities tailored to each district. Similar targeted approaches are likely necessary in other settings, which may affect the cost-effectiveness in any particular setting. Further, the costs presented here do not account for potential changes to costs structures, demand for services, and average unit costs over time. In the future, increased uptake of family planning, further increases in demand for and use of services, and so forth will likely change the unit costs of delivering MNH services as well as the mix of activities needed. Thus, the cost-effectiveness of district health strengthening approaches such as SMGL will likely also change over time.

The SMGL project could be paid for by increasing health spending from the 7.3% of GDP in 2015 (in Uganda) and 5.4% (in Zambia) to 7.5% and 5.8% of GDP, respectively.

This study adds to the literature by presenting actual costs and effects of a health systems strengthening approach that addressed the 3 key barriers to receiving MNH care. We find that the approach costs about $177 to $206 per year of life gained, depending on the context. Ministries of Health and donor agencies have already demonstrated a willingness to pay this amount per year of life gained; for example, first-line antiretroviral therapy cost over $200 per person per year across 5 countries (including Zambia) in sub-Saharan Africa in 2010.^50^ Thus, we conclude that the SMGL approach as demonstrated likely represents a very cost-effective health investment.

## Supplementary Material

Supplement 1

## Notes

**Peer Reviewed**

**Funding:** Saving Mothers, Giving Life implementation was primarily funded by the Office of the Global AIDS Coordinator, USAID, Washington, DC; US Centers for Disease Control and Prevention, Atlanta, Georgia (Cooperative agreements GPS003057 and GPS002918); Merck for Mothers; and Every Mother Counts. This study was funded by USAID through the Health Finance and Governance Project (Cooperative Agreement No: AID-OAA-A-12-00080). The funding agencies had no influence or control over the content of this article.
