# Supplementary material for: The Costs and Cost-Effectiveness of a District-Strengthening Strategy to Mitigate the 3 Delays to Quality Maternal Health Care: Results From Uganda and Zambia
Source: Glob Health Sci Pract. 2019 Mar 11;7(Suppl 1):S104–22. doi: 10.9745/GHSP-D-18-00429 (PMC6519668; doi:10.9745/GHSP-D-18-00429)
Supplement: Supplement 1 [file GHSP-D-18-00429_index.html]

Supplement to The Costs and Cost-Effectiveness of a District-Strengthening Strategy to Mitigate the 3 Delays to Quality Maternal Health Care: Results From Uganda and Zambia | Global Health: Science and Practice

## Supplemental material

**Files in this Data Supplement:**

- Data Sources and Adjustments for Secular Trends - Text s01, PDF
