# Supplementary material for: The Costs and Cost-Effectiveness of a District-Strengthening Strategy to Mitigate the 3 Delays to Quality Maternal Health Care: Results From Uganda and Zambia
Source: Glob Health Sci Pract. 2019 Mar 11;7(Suppl 1):S104–22. doi: 10.9745/GHSP-D-18-00429 (PMC6519668; doi:10.9745/GHSP-D-18-00429)
Supplement: Supplement 1 [file 18-00429-Johns-Supplement1.pdf]

Johns B, Hangoma P, Atuyambe L, et al; Saving Mothers, Giving Life Working Group. The costs and cost-effectiveness of a district-strengthening strategy to mitigate the 3 delays to quality maternal health care: results from Uganda and Zambia. *Glob Health Sci Pract*. 2019;7(suppl 1). https://doi.org/10.9745/GHSP-D-18-00429

SUPPLEMENT 1. Data Sources and Adjustments for Secular Trends

Uganda

Maternal mortality ratio (MMR): In the Demographic and Health Surveys (DHS), the MMR was estimated at 440 maternal deaths per 100,000 live births in 2011 and 336 maternal deaths per 100,000 live births in 2016, or 15.98% lower in 2016 than in 2012.[27,28] The MMR in the 4 Saving Mothers, Giving Life (SMGL) districts in 2012 was measured through SMGL Reproductive Age Mortality Survey to be 534 per 100,000 live births in 2012.[30] To account for secular trends, we applied the decline observed between the 2 DHS surveys to the observed MMR in the SMGL districts in 2012. Conservatively, we adjusted this baseline MMR downward by 15.98% to 449 per 100,000 live births.

Perinatal mortality rate (PMR): The perinatal mortality rate measured in the 2 DHS was well within the sampling margin of error, and provided no conclusive evidence that any significant declines in the PMR occurred over the time period of 2011–2016.[27,28] Thus, we did not adjust the PMR in Uganda for secular trends.

Zambia

MMR: Unlike Uganda, Zambia does not have DHS that closely matched in time the SMGL program (the latest were conducted in 2007 and 2013). Thus, we used published estimates of trends in MMR.[42] However, projections for 2016 were not available for this paper. From 2011 to 2015, the MMR declined by an average of 2.85% per year; we used the 2.85% decline to project the MMR in 2016. The MMR from global estimates was 243 per 100,000 live births in 2012 in Zambia, and we estimated it was 218 per 100,000 live births in 2016. In the 4 SMGL districts, the MMR was 370 per 100,000 live births in 2012. To account for secular trends, we adjusted this to 331.5 per 100,000 live births, following the method described for Uganda.

PMR: Unlike for MMR, there are no published projections for PMR. The DHS from 2007 and 2013 indicated no difference in the stillbirth rate but detected declines in the newborn mortality rate.[25,26] To account for secular trends in the newborn mortality rate, we used projections of neonatal mortality rates as an imperfect proxy.[43] From 2012 to 2016, neonatal mortality declined by 9.92% in Zambia. The 9.92% decline was applied to the newborn mortality portion of the 2012 PMR in the 4 SMGL districts, and the observed PMR of 37.9 was adjusted to 37.2 for secular trends.
